# PRIMIR on Tone

**DOI:** 10.3389/fpsyg.2018.01007

**Published:** 2018-06-15

**Authors:** Suzanne Curtin, Janet F. Werker

**Affiliations:** ^1^Department of Psychology, University of Calgary, Calgary, AB, Canada; ^2^Department of Psychology, The University of British Columbia, Vancouver, BC, Canada

**Keywords:** PRIMIR, tone, speech perception, language acquisition, word learning, bilingualism, infancy

Processing Rich Information from Multidimensional Interactive Representations (PRIMIR) was first developed to address divergent findings in the literature surrounding early speech perception and word learning (Werker and Curtin, 2005; Curtin and Werker, 2007). Specifically, there was a controversy in the literature at the time indicating that while under some circumstances infants and toddlers are able to discriminate a mispronunciation of a known word, in other testing circumstances, they had difficulty using phonetic/phonological differences to guide word learning. PRIMIR was developed as a framework for understanding why infants attend to some kinds of information over others in different testing situations. Integral to PRIMIR are the representations and processing that simultaneously impact how the infant interprets the information in the signal. PRIMIR assumes infants have access to a rich signal containing multi-sensory information. It further assumes that infants have initial biases that work together with learning mechanisms (e.g., statistical, comparison/contrast) to help process, organize, and store the information gleaned from the signal. Information is stored within emergent interactive representational planes: general perceptual, word form, and phoneme, and is processed using three dynamic filters: initial biases (e.g., preferences for speech, native-language rhythm, and infant-directed speech), the developmental level of the infant, and the task that the child is facing (e.g., discriminating sounds, learning words). While the initial biases draw the infant's attention to the linguistic signal, the developmental level and the task will influence what information is attended to, stored, and used at any given time. That is, the states of the infant's various representations and the task itself will shift how the infant interprets the information. With this in mind, rather than clear-cut developmental boundaries of when an infant might show evidence of acquiring any particular linguistic ability, the process is more multiplexed, with the use of some types of information potentiated only once the relevant representations are in place. While the input that the infant has access to is rich, some aspects of the signal are inherently more salient than others, and some of those properties that are perhaps not as inherently salient can become enhanced through experience, development, and their contribution to category formation. In other words, some sound properties have raw acoustic salience, while others require converging sources of evidence (be it multi-sensory or contextual) to be processed, discriminated, or learned. Tone is a paradigmatic example of how acoustic salience (pitch), and the representational structures that are in place influence its processing.

In considering the interface of initial perceptual biases, the extraction of word forms and connecting them to concepts, and the emergence of phonemes, PRIMIR initially focused on segments as both carriers of linguistic contrast (e.g., /dag/ “dog” vs. /bag/ “bog”) and of indexical cues, such as talker and affect. However, pitch is perhaps an even more telling example of a perceptual property that can both be implemented as a tone (carried on vowels, voiced/sonorant consonants, or syllables) to designate and contrast meaning as well as an indexical signal of an array of functions from focus to emotional valence.The range of articles in this special issue eloquently describe these multiple functions and address the unique challenges for learners. We highlight some of these findings to illustrate how PRIMIR can help explain how infants simultaneously learn to use pitch for indexical information and tone for word learning.

Consistent with PRIMIR are a number of studies examining the discrimination and functional use of native and non-native tones. In some studies findings are consistent with how phonetic sensitivities develop with the general perceptual plane. For example, both babies growing up in tone and non-tone languages discriminate tones at a younger age (4–6 months), and stop doing so by 9-months unless they grow up in a tone language (e.g., Mattock and Burnham, 2006; Yeung et al., 2013; Liu and Kager, 2014). However, also consistent with PRIMIR there are variations in performance as a function of the discrimination procedure (Gotz et al., this issue), and as a function of the acoustic salience of the tones being compared (Chen et al., this issue; Cheng and Lee, this issue; Tsao, this issue). A finding in the tone literature that uniquely supports PRIMIR, is that even in the cases where there is a decline in non-native tone discrimination between 6- and 9-months, there is a rebound in discrimination by 18-months (Gotz et al., this issue; Liu and Kager, this issue) but an inability to use these (non-native) tone distinctions to guide word learning at 18 months (Burnham et al., this issue; Liu and Kager, this issue). These findings reveal simultaneous access to multiple representational planes. Importantly, within the PRIMIR framework, emergent phonemes help to direct information about lexical processing around 18 months of age. Thus, while infants of this age can still access acoustic information contained within the General Perceptual plane and direct attention in a discrimination task (as seen in the rebound in tone discrimination), the native phonological system constrains the mapping of non-native contrasts to distinct words further suggesting that by 18 months, infants treat lexical tones as phonemic elements. In our refocused version of PRIMIR (Curtin et al., 2011), we began to explore how bilingual infants' experiences with their dual language input may result in them approaching tasks differently than monolingual peers. We had not yet considered, however, that their dual language experience might boost their attention to acoustic and phonetic information in tone. Nor have we considered what type of dual language experience (e.g., tone/non-tone, two non-tone) might boost attention. Bilingual infants learning two non-tone languages show a rebound in tone discrimination 6 months earlier than monolingual infants (Liu and Kager, 2017). Bilingual English-Mandarin infants demonstrate an ability to use the acoustically salient Thai tone contrast in a word learning task while monolingual Mandarin infants do not (Burnham et al., this issue). The extent to which dual language experience shifts infants' use of the dynamic filters across the representational planes is an exciting new direction for the further development of PRIMIR.

## Author contributions

All authors listed have made a substantial, direct and intellectual contribution to the work, and approved it for publication.

### Conflict of interest statement

The authors declare that the research was conducted in the absence of any commercial or financial relationships that could be construed as a potential conflict of interest.
